# Factors Associated With Persistent Opioid Use Among Injured Workers’ Compensation Claimants

**DOI:** 10.1001/jamanetworkopen.2018.4050

**Published:** 2018-10-26

**Authors:** Nathan N. O’Hara, Andrew N. Pollak, Christopher J. Welsh, Lyndsay M. O’Hara, Alyson K. Kwok, Alexandra Herman, Gerard P. Slobogean

**Affiliations:** 1 University of Maryland School of Medicine, Baltimore; 2 Glenrose Rehabilitation Hospital, Edmonton, Alberta, Canada

## Abstract

**Importance:**

There is a paucity of data on persistent opioid use and factors associated with persistent opioid use among workers’ compensation claimants.

**Objective:**

To determine the proportion of injured workers who filled an opioid prescription beyond 90 days from injury and the factors associated with persistent opioid use among workers’ compensation claimants.

**Design, Setting, Participants:**

This retrospective cohort study collected workers’ compensation claims data from January 1, 2008, to December 31, 2016, from the Chesapeake Employers’ Insurance Company in Maryland. All workers’ compensation claimants injured during the study years and with at least 1 filled opioid prescription were eligible for inclusion. For patients who had unique injury claims in multiple years of the study, only the first claimed injury was included in our analysis. Patients who died as a result of the claimed injury were excluded. The analysis was performed between October 2017 and August 2018.

**Main Outcomes and Measures:**

The primary outcome was persistent opioid use, defined as an opioid prescription fulfillment beyond 90 days from the date of injury. Multivariable regression was used to determine prognostic factors of persistent opioid use.

**Results:**

Of the 9596 study participants (mean [SD] age, 43 [12.3] years; 6218 [65.1%] male), 2741 (28.6%) filled an opioid prescription more than 90 days from their date of injury. Participants aged 60 years or older (odds ratio [OR], 1.92; 95% CI, 1.56-2.36), crush injuries (OR, 1.55; 95% CI, 1.28-1.89), strain and sprain injuries (OR, 1.54; 95% CI, 1.36-1.75), annual income more than $60 000 (OR, 1.31; 95% CI, 1.07-1.61), and concomitant diagnoses for chronic joint pain (OR, 1.98; 95% CI, 1.79-2.20) were significantly associated with persistent opioid use. Compared with workers with claims designated as permanent partial disability, workers with medical-only claims were significantly less likely to have persistent opioid use at 90 days postinjury (OR, 0.17; 95% CI, 0.15-0.20).

**Conclusions and Relevance:**

A high proportion of persistent opioid use was observed in this workers’ compensation cohort. Interventions to lower persistent opioid use in this population should target patients with the characteristics identified in this study.

## Key Points

**Question:**

What was the proportion of persistent opioid use and the patient-level factors associated with persistent opioid use among workers’ compensation claimants?

**Findings:**

In this cohort study of 9596 workers’ compensation claimants who were initially treated with an opioid prescription, approximately 30% of claimants continued to fill opioid prescriptions beyond 90 days from injury. Baseline characteristics, including increased age, preinjury income more than $60 000, crush injuries, strain or sprain injuries, and a concomitant diagnosis of chronic joint pain were associated with persistent opioid use.

**Meaning:**

The findings suggest workers’ compensation claimants have a high proportion of persistent opioid use. Interventions to lower persistent opioid use among this population should target patients with the identified factors, and since persistent opioid use does not correlate well with injury severity, consideration should be given to not initiating opioid use for nonsevere injuries.

## Teaser

This cohort study examines the proportion of injured workers’ compensation claimants who filled an opioid prescription beyond 90 days from their time of injury and the factors associated with persistent opioid use.

## Introduction

In 2016, more than 11.5 million Americans aged 12 years or older (4.4% of the population) reported misuse of prescription opioids and more than 14 000 people died from overdoses involving prescription opioids in the United States.^1,2^ Despite these known risks and very little evidence to support long-term opioid therapy for pain management, at least 214 million opioid prescriptions were dispensed every year in the United States since 2006.^3,4^ In response, the US Department of Health and Human Services declared a public health emergency in October 2017 to address the national opioid crisis.^5^

The number of opioid prescriptions per workers’ compensation claim in the United States has also climbed considerably since 2003.^6^ There is a strong economic rationale for studying persistent opioid use among injured workers. Persistent opioid use in injured workers were previously linked with more costly claims and attributed to an overall loss in work productivity.^7,8^ The factors that led to persistent opioid use or dependence are unknown. This study of workers’ compensation records may provide a unique insight into 18 to 65 years of age group most at risk for an opioid misuse, addiction, or overdose.^9^

The objective of this study was to determine the proportion of injured workers who filled an opioid prescription beyond 90 days from their time of injury and the factors associated with persistent opioid use among injured workers’ compensation claimants in Maryland. By investigating persistent opioid use, a broad public health concern, under the unique financial protection structure of workers’ compensation claims, we sought to understand the factors that may contribute to the use of long-term prescription opioid use among a high-risk group of patients. We do not aim to present an exhaustive list of all the factors associated with persistent opioid in this population but rather aim to determine the extent to which factors that are routinely available in administrative databases are associated with persistent opioid use in injured workers. We hypothesized that persistent opioid use after a workers’ compensation injury would be associated with increased age, lower annual income, and claims owing to a sprain or strain injury. The association between persistent opioid use and increased age is supported by previous research on opioid-naive surgical patients.^10^ The association of persistent opioid use with lower annual income and strain and sprain injuries was demonstrated in a study involving Australian workers’ compensation claimants.^11^

## Methods

### Data Sources and Patient Cohort

This retrospective cohort study examined workers’ compensation claims data from the Chesapeake Employers’ Insurance Company to determine factors associated with persistent opioid use among injured workers’ compensation claimants. The Chesapeake Employers’ Insurance Company is the largest writer of workers’ compensation insurance in Maryland, insuring approximately 266 000 workers in 2016. All claims from January 1, 2008, to December 31, 2016, were included. For patients who had unique injury claims in multiple years of the study, only the first claimed injury was included in our analysis. Patients who died as a result of the claimed injury were excluded. To be included in the analysis patients must have filled at least 1 opioid prescription within 90 days of the injury for which the claim was submitted. Prescribed opioids were determined based on workers’ compensation reimbursements. This study was approved by the University of Maryland Institutional Review Board with a waiver of consent. The analysis and reporting followed the Strengthening the Reporting of Observational Studies in Epidemiology (STROBE) reporting guideline and the Transparent Reporting of a Multivariable Prediction Model for Individual Prognosis or Diagnosis (TRIPOD) reporting guideline.^12,13^

### Study Outcomes

The primary outcome of the study was persistent opioid use, defined as a filled opioid prescription paid for by the workers’ compensation claim more than 90 days from the date of injury. Ninety days from the time of injury was selected as the study’s definition for persistent use and was consistent with the recent publications on persistent opioid use.^14,15^ Secondary outcomes included filled opioid prescriptions more than 180 and 365 days from the date of injury. For a patient to be categorized as a persistent opioid user at 180 days postinjury, the patient must have claimed at least 1 opioid prescription within 90 days of injury and at least 1 opioid prescription after 180 days from injury. Similarly, for a patient to be categorized as a persistent opioid users at 365 days postinjury, the patient must have claimed at least 1 opioid prescription within 90 days of injury and at least 1 opioid prescription after 365 days from injury. Only the first injury claim for a participant during the study period was included in our analysis and subsequent claims by a participant were excluded from the analysis. If a participant had a subsequent injury claim during the study period for which they filled an opioid prescription, we assumed that prescription to be associated with the second claim and it would therefore not count toward persistent opioid use for the participant’s claim that was included in the analysis.

### Prognostic Factors

Candidate factors associated with persistent opioid use included sociodemographic and clinical covariates from the insurance claims data. Demographic characteristics included age and sex. *International Classification of Diseases, Ninth Revision* (*ICD-9*) codes documented on the insurance claims were used to determine the type of injury.^16^ The disability status was categorized based on the most severe level of claim, as adjudicated by the Maryland Workers’ Compensation Commission. The levels included medical-only claims, as well as 4 disability levels ranging from temporary partial disability to permanent total disability. The employment type was categorized using the Standard Occupational Classifications and the study participants’ employer was coded as the state or a private company.^17^ The number of years with the current employer, the annual income of the study participants, the number of days away from work owing to the injury, and full-time employment status was extracted from the claims data. Surgical procedures were identified based on claimed *Current Procedural Terminology* codes. The Clinical Classification System of the Agency for Healthcare Research and Quality was used to categorize mental health diagnosis based on *ICD-9* and *International Classification of Diseases, Tenth Revision* codes.^18^ These codes were combined into several categories including anxiety, mood disorders, suicidality, personality disorders, schizophrenia, and substance use disorders. Additional pain diagnoses specific to back, neck, joint, and other regions were also recorded based on *ICD-9* codes (eTable in the Supplement). Missing values for age and annual income composed less than 10% of the data and were imputed using multiple imputation.^19^

### Statistical Analysis

After initial bivariate testing of the association between prognostic factors and persistent opioid use more than 90 days postinjury were performed using χ^2^ tests for categorical data and *t* tests for continuous data, 3 separate prediction models were developed. The primary analysis determined factors that were prognostic of a filled opioid prescription more than 90 days postinjury. Two additional models were developed to determine prognostic factors for a filled opioid prescription more than 180 and 365 days postinjury. Backward elimination stepwise modeling based on a minimum Akaike information criterion with whole effects was used to select factors associated with inclusion in the final multivariable logistic regression models. Discriminatory ability was determined using the area under the curve (AUC) and receiving operating characteristic curve statistics. Calibration of the models was assessed using the Hosmer-Lemeshow χ^2^ test.^20^ The models were internally validated using bootstrap resampling with replacement for 200 iterations.^21^ Two-tailed α = .05 was considered statistically significant. The measure of association between the included factors and the dependent variables are reported as adjusted β coefficients with standard errors and adjusted odds ratios (ORs) with 95% confidence intervals. All statistical analyses were performed using JMP Pro, version 13 (SAS Institute).

## Results

The study included 100 312 unique claims registered with the Chesapeake Employers’ Insurance Company between January 1, 2008, and December 31, 2016. Of these claims, 89 007 study participants were excluded from the data for not having a single opioid prescription filled as part of their insurance claim. Claims pertaining to a second injury during the study period excluded an additional 1709 claimed injuries. The remaining 9596 study participants (mean [SD] age, 43 [12.3] years; 6218 [65.1%] male) were included for analysis.

Descriptive statistics of participant characteristics are displayed in Table 1. Of the 9596 study participants, 2741 study participants (28.6%) filled an opioid prescription more than 90 days from their date of injury; 1762 (18.4%) filled an opioid prescription more than 180 days from their date of injury; and 902 (9.4%) filled an opioid prescription more than 1 year from their date of injury. It should also be noted that 83 862 of the overall claimants (77%) did not have a single opioid prescription filled as part of their claim.

**Table 1.** Characteristics of the Included Patients and Stratified by Persistent Opioid Use

Variable	No. (%)			*P* Value
	All Patients	Opioid Prescription >90 d from Injury	No Opioid Prescription Beyond 90 d From Injury	
No.	9596	2741	6855	
Age, y				
15-29	1642 (17.1)	294 (10.7)	1348 (20.0)	<.001
30-39	2144 (22.3)	576 (21.0)	1568 (22.9)	
40-49	2646 (27.5)	890 (32.5)	1752 (25.6)	
50-59	2310 (24.1)	718 (26.2)	1592 (23.2)	
≥60	858 (8.9)	263 (9.6)	595 (8.7)	
Male	6218 (65.1)	1800 (65.9)	4417 (64.8)	.33
Type of injury				
Soft-tissue or contusion	3753 (39.1)	906 (33.1)	2847 (41.5)	<.001
Strain or sprain	2444 (25.4)	739 (27.0)	1705 (24.9)	
Open wound	1542 (16.1)	494 (18.0)	1048 (15.3)	
Fracture	667 (7.0)	224 (8.2)	443 (6.5)	
Crush	632 (6.6)	216 (7.9)	416 (6.1)	
Poisoning^^a^^	427 (4.5)	122 (4.5)	305 (4.5)	
Other or unspecified	131 (1.4)	40 (1.5)	91 (1.3)	
Disability status				
Permanent total disability	11 (0.1)	9 (0.33)	2 (0.03)	<.001
Permanent partial disability	3712 (38.7)	1675 (61.1)	2037 (29.7)	
Temporary total disability	2704 (28.2)	727 (26.5)	1977 (28.8)	
Temporary partial disability	128 (1.3)	51 (1.9)	77 (1.1)	
Medical only	3041 (31.7)	279 (10.2)	2762 (40.3)	
Type of occupation				
Operatives and technicians	3111 (32.4)	940 (34.3)	2717 (31.7)	<.001
Service workers	2680 (27.9)	767 (28.0)	1913 (27.9)	
Laborers and helpers	1693 (17.6)	499 (18.2)	1194 (17.4)	
Professionals	864 (9.0)	219 (8.0)	645 (9.4)	
Office workers	849 (8.8)	231 (8.4)	618 (9.0)	
Sales workers	330 (3.4)	65 (2.4)	265 (3.9)	
Unrecorded	69 (0.7)	20 (0.7)	49 (0.7)	
Employer				
Private company	6549 (68.2)	1875 (68.4)	4674 (68.2)	.83
State employed	3047 (31.8)	866 (31.6)	2181 (31.8)	
Current employment, median (IQR), y	2.7 (0.7-8.5)	3.3 (0.8-10.0)	2.5 (0.7-7.9)	<.001
Annual preinjury income, $				
<20 000	1036 (10.8)	328 (12.0)	708 (10.3)	<.001
20 000-39 999	5177 (54.0)	1175 (42.9)	4002 (58.4)	
40 000-59 999	2324 (24.4)	827 (30.2)	1497 (21.8)	
≥60 000	1059 (11.1)	411 (15.0)	648 (9.5)	
Full-time employment	7538 (78.6)	2099 (76.6)	5439 (79.3)	.003
Surgical treatment	1325 (13.8)	290 (10.6)	1035 (15.1)	<.001
Chronic pain diagnosis^^b^^				
Joint	3616 (37.7)	1447 (52.8)	2169 (31.6)	<.001
Back	1633 (17.0)	504 (18.4)	1129 (16.5)	.02
Other	822 (8.6)	411 (15.0)	411 (6.0)	<.001
Neck	820 (8.5)	276 (10.1)	544 (7.9)	<.001
Concomitant mental health diagnosis				
Anxiety	78 (0.8)	15 (0.6)	63 (0.9)	.07
Substance use	60 (0.6)	16 (0.6)	44 (0.6)	.74
Mood disorder	27 (0.3)	8 (0.3)	19 (0.3)	.90

Abbreviation: IQR, interquartile range.

^a^ *International Classification of Diseases, Ninth Revision* codes used to determine poisoning injuries were classified as poisoning by either unspecified gas and vapor, or by agents primarily affecting the skin. It is therefore assumed not be related to an opioid overdose.

^b^ Includes common *International Classification of Diseases, Ninth Revision* codes

In our bivariate analysis, persistent opioid use was associated with increased age, more years with the current employer, a higher annual income, part-time employment, and less surgical treatment (Table 1). We observed significant differences in persistent opioid use by type of injury, type of occupation, and disability status. Persistent opioid use was associated with a concomitant pain diagnosis.

Multivariable models for factors associated with persistent opioid use at 90, 180, and 365 days postinjury are displayed in Table 2. In the primary model, study participants with a chronic joint pain diagnosis were twice as likely to fill an opioid prescription beyond 90 days from injury than those who did not have such a diagnosis (OR, 1.98; 95% CI, 1.79-2.20). Participants with other chronic pain diagnoses such as migraines, headaches, or fibromyalgia were 3 times more likely to fill an opioid prescription beyond 90 days from injury than those who did not have other chronic pain diagnoses (OR, 2.96; 95% CI, 2.51-3.48). Compared with injured workers younger than 30 years, those aged 30 to 39 years (OR, 1.43; 95% CI, 1.20-1.69), 40 to 49 years (OR, 1.89; 95% CI, 1.61-2.22), 50 to 59 years (OR, 1.83; 95% CI, 1.55-2.17), and 60 years or older (OR, 1.92; 95% CI, 1.56-2.36) were more likely to use opioids consistently. Workers with greater annual preinjury incomes were more likely to have persistent opioid use at 90 days postinjury. Workers without full-time employment were more likely to have persistent opioid use 90 days postinjury (OR, 1.23; 95% CI, 1.09-1.39). Claimants with annual preinjury incomes of at least $60 000 were more likely to have persistent opioid use relative to those with annual preinjury incomes less than $20 000 (OR, 1.31; 95% CI, 1.07-1.61). Compared with workers with claims designated as permanent partial disability, workers with medical-only claims were significantly less likely to have persistent opioid use at 90 days postinjury (OR, 0.17; 95% CI, 0.15-0.20). Furthermore, crush injuries (OR, 1.55; 95% CI, 1.28-1.89), strain and sprain injuries (OR, 1.54; 95% CI, 1.36-1.75), and open wound injuries (OR, 1.34; 95% CI, 1.16-1.54) were more likely to be associated with persistent opioid use when compared with soft-tissue and contusion injuries.

**Table 2.** Multivariable Logistic Regression Models of Persistent Opioid Use at 90, 180, and 365 Days Postinjury

Variables	90 d Postinjury^a^^,^^b^		180 d Postinjury^b^^,^^c^		365 d Postinjury^b^^,^^d^	
	Adjusted β (SE)	Adjusted OR (95% CI)	Adjusted β (SE)	Adjusted OR (95% CI)	Adjusted β (SE)	Adjusted OR (95% CI)
Age, y						
15-29	0 [Reference]	1 [Reference]	0 [Reference]	1 [Reference]	0 [Reference]	1 [Reference]
30-39	0.36 (0.09)^e^	1.43 (1.20-1.69)^e^	0.32 (0.10)^e^	1.37 (1.12-1.67)^e^	0.22 (0.14)	1.25 (0.96-1.63)
40-49	0.64 (0.08)^e^	1.89 (1.61-2.22)^e^	0.54 (0.10)^e^	1.71 (1.42-2.07)^e^	0.50 (0.13)^e^	1.65 (1.28-2.12)^e^
50-59	0.61 (0.09)^e^	1.83 (1.55-2.17)^e^	0.47 (0.10)^e^	1.60 (1.32-1.95)^e^	0.41 (0.13)^e^	1.51 (1.16-1.97)^e^
≥60	0.65 (0.11)^e^	1.92 (1.56-2.36)^e^	0.58 (0.12)^f^	1.78 (1.40-2.27)^e^	0.52 (0.16)^e^	1.67 (1.21-2.32)^e^
Type of injury						
Soft-tissue or contusion	0 [Reference]	1 [Reference]	0 [Reference]	1 [Reference]	0 [Reference]	1 [Reference]
Strain or sprain	0.43 (0.06)^e^	1.54 (1.36-1.75)^e^	0.47 (0.07)^e^	1.60 (1.39-1.85)^e^	0.43 (0.10)^e^	1.54 (1.27-1.86)^e^
Open wound	0.29 (0.07)^e^	1.34 (1.16-1.54)^e^	0.32 (0.08)^e^	1.38 (1.17-1.62)^e^	0.21 (0.11)	1.24 (1.00-1.54)
Fracture	0.19 (0.10)	1.20 (0.99-1.46)	0.23 (0.11)^f^	1.25 (1.01-1.56)^f^	0.32 (0.14)^f^	1.38 (1.05-1.81)^f^
Crush	0.44 (0.10)^e^	1.55 (1.28-1.89)^e^	0.48 (0.11)^e^	1.61 (1.29-2.01)^e^	0.37 (0.15)^f^	1.45 (1.08-1.94)^f^
Poisoning^g^	0.23 (0.12)	1.25 (0.95-1.60)	0.39 (0.14)^e^	1.48 (1.13-1.94)^e^	0.25 (0.18)	1.30 (0.90-1.86)
Other or unspecified	0.50 (0.21)^f^	1.66 (1.09-2.52)^f^	0.53 (0.24)^f^	1.70 (1.06-2.73)^f^	0.78 (0.29)^e^	2.18 (1.23-3.85)^e^
Disability status						
Permanent total disability	1.51 (0.81)	4.52 (0.92-22.17)	2.10 (0.81)^e^	8.016 (1.66-40.07)^f^	1.83 (0.67)^e^	6.29 (1.68-23.57)^e^
Permanent partial disability	0 [Reference]	1 [Reference]	0 [Reference]	1 [Reference]	0 [Reference]	1 [Reference]
Temporary total disability	−0.67 (0.06)^e^	0.51 (0.45-0.57)^e^	−0.67 (0.07)^e^	0.51 (0.45-0.58)^e^	−0.42 (0.09)^e^	0.66 (0.55-0.78)^e^
Temporary partial disability	−0.24 (0.19)	0.78 (0.54-1.14)	0.01 (0.20)	1.02 (0.70-1.51)	−0.02 (0.26)	0.98 (0.58-1.64)
Medical only	−1.78 (0.08)^e^	0.17 (0.15-0.20)^e^	−1.94 (0.10)^e^	0.14 (0.12-0.17)^e^	−1.62 (0.14)^e^	0.20 (0.16-0.26)^e^
Occupation						
Operatives and technicians	^h^	^h^	^h^	^h^	0 [Reference]	1 [Reference]
Service workers	^h^	^h^	^h^	^h^	0.02 (0.10)	1.02 (0.85-1.24)
Labors and helpers	^h^	^h^	^h^	^h^	0.16 (0.11)	1.17 (0.95-1.46)
Professionals	^h^	^h^	^h^	^h^	−0.15 (0.15)	0.86 (0.63-1.16)
Office workers	^h^	^h^	^h^	^h^	0.12 (0.14)	1.13 (0.86-1.49)
Sales workers	^h^	^h^	^h^	^h^	−0.25 (0.25)	0.78 (0.48-1.26)
Unrecorded	^h^	^h^	^h^	^h^	−1.16 (0.72)	0.32 (0.08-1.31)
Annual preinjury income, $						
<20 000	0 [Reference]	1 [Reference]	0 [Reference]	1 [Reference]	0 [Reference]	1 [Reference]
20 000-39 999	−0.01 (0.08)	0.99 (0.85-1.16)	−0.04 (0.09)	0.97 (0.80-1.15)	0.09 (0.13)	1.10 (0.85-1.41)
40 000-59 999	0.16 (0.09)	1.18 (0.99-1.40)	0.07 (0.10)	1.07 (0.89-1.30)	0.34 (0.13)^f^	1.40 (1.08-1.82)^f^
≥60 000	0.27 (0.10)^e^	1.31 (1.07-1.61)^e^	0.24 (0.11)^f^	1.28 (1.03-1.59)^f^	0.39 (0.15)^e^	1.48 (1.11-1.99)^e^
Employment type						
Full time	0 [Reference]	1 [Reference]	^h^	^h^	^h^	^h^
Non–full time	0.21 (0.06)^e^	1.23 (1.09-1.39)^e^	^h^	^h^	^h^	^h^
Surgical treatment	^h^	^h^	^h^	^h^	−0.23 (0.14)	0.79 (0.60-1.04)
Chronic pain diagnosis						
Joint	0.68 (0.5)^e^	1.98 (1.79-2.20)^e^	0.57 (0.06)^e^	1.77 (1.58-2.00)^e^	0.68 (0.08)^e^	1.97 (1.67-2.32)^e^
Other	1.08 (0.08)^e^	2.96 (2.51-3.48)^e^	1.23 (0.08)^e^	3.43 (2.90-4.08)^e^	1.51 (0.10)^e^	4.53 (3.71-5.53)^e^
Back pain	^h^	^h^	^h^	^h^	0.38 (0.09)^e^	1.46 (1.22-1.74)^e^
Substance abuse diagnosis	0.56 (0.30)	1.76 (0.97-3.19)	^h^	^h^	^h^	^h^
Intercept	−1.53 (0.11)^e^		−2.01 (0.12)^e^		−3.45 (0.22)^e^	

Abbreviation: OR, odds ratio.

^a^ Area under the receiver operating characteristic curve = 0.75.

^b^ β coefficients and ORs are adjusted for all other covariates displayed for a given model.

^c^ Area under the receiver operating characteristic curve = 0.75.

^d^ Area under the receiver operating characteristic curve = 0.76.

^e^ *P* < .01.

^f^ *P* < .05.

^g^ *International Classification of Diseases, Ninth Revision *codes used to determine poisoning injuries were classified as poisoning either by unspecified gas and vapor or by agents primarily affecting the skin. It is therefore assumed not be related to an opioid overdose.

^h^ These variable were not selected for inclusion in the model based on the stepwise technique with a minimum Akaike information criterion.

The association between persistent opioid use and age, type of injury, disability status, preinjury income, and chronic joint pain remained fairly consistent across all 3 models (90, 180, and 365 days postinjury). However, in the 365-day model, the strength of the association of persistent opioid use among participants with other chronic pain diagnosis was greater (OR, 4.53; 95% CI, 3.71-5.53). Unique to the 365-day model, a chronic back pain diagnosis increased the likelihood of persistent opioid use (OR, 1.46; 95% CI, 1.22-1.74) and surgical treatment for the injury was nominally protective against persistent opioid use (OR, 0.79; 95% CI, 0.60-1.04).

All models demonstrated similar discriminative ability, with the 90- and 180-day models having an AUC of 0.75 and the 365-day model an AUC of 0.76. Close agreement between derivation models and the bootstrap corrected AUC was observed for the 90-day (AUC, 0.75), 180-day (AUC, 0.76), and 365-day (AUC, 0.74) models.

## Discussion

In this study of workers’ compensation injuries in Maryland, 2741 claimants (28.6%) with an initial opioid prescription filled at least 1 opioid prescription more than 90 days from the time of injury. Nearly 10% of the injured workers filled an opioid prescription beyond 365 days from their date of injury. Persistent opioid use was significantly associated with increased age, preinjury incomes of $60 000 or more, claims adjudicated as permanent total disability, and a concomitant diagnosis of chronic joint pain or another pain diagnosis such as migraines or fibromyalgia. Claimants with crush injuries and strain or sprain injuries were 50% more likely than those with soft-tissue or contusion injuries to have persistent opioid use.

The proportion of injured workers with persistent opioid use substantially exceeds recent reports on surgical patients at 90 days (28.6% vs 6.0%) and the national rate at 1-year from initial therapy reported by the Centers for Disease Control and Prevention.^15,22^ However, it should be noted that the surgical study excluded patients with opioid prescriptions in the year preceding their surgical treatment, which likely accounts for some of the discrepancy.^15^ The predictive models in this study suggest several unique characteristics of the workers’ compensation population in Maryland that may help explain why their proportion of persistent opioid use largely exceeds that of previous studies. These factors include a high prevalence of chronic joint pain diagnosis along with the high proportion of strain and sprain injuries that may lack definitive resolution.^23^

This study builds on previous opioid use research in workers’ compensation claimants with its broad inclusion criteria and substantially longer study duration. The inclusion of the adjudicated level of benefits to the claimant ranging from medical-only claims to permanent total disability is novel.^7^ Beyond insight into the severity of the injury, the benefit level of the claimant may provide a surrogate marker for the motivation of the worker to return to work and the legal representation available to the worker to pursue more substantial benefits. This is evident by the significant protective effect of medical-only claims and temporary total disability claims when compared with permanent partial disability claims and the increased likelihood of persistent opioid use among permanent total disability claims. The current structure of adjudication and compensation for work-related injuries in Maryland may unintentionally encourage persistent opioid use. The design of the Maryland Workers’ Compensation system is to allow disputes over medical issues to be adjudicated in a legal setting. The decisions may reflect the nature of the medical supporting documents and presentation as the basis for a legal decision rather than an accurate reflection of the medical condition. Continued prescriptions for pain management can be used to legally support a continued injury claim. Also of note is the strong association between unspecific types of injuries and persistent opioid use. A lack of specificity in diagnosis may prolong delays in treatment, recovery, and return to work.

Many of our findings were consistent with previous research. Patients with a chronic joint pain diagnosis were more likely to be persistent opioid users.^15,24,25^ Previous studies have reported an association between persistent opioid use and a back pain diagnosis.^15,25,26^ In the current study, participants with back pain were 46% more likely to use opioid beyond 1 year from injury (OR, 1.46; 95% CI, 1.22-1.74). The type of occupation (eg, laborers and services workers) has been previously linked with long-term opioid use.^27^ We did not observe an association between service workers and persistent opioid use but did find an association between laborers and opioid use beyond 1 year. Consistent with other studies, increased age was associated with persistent opioid use.^10,25^ The association between higher preinjury income and persistent opioid use was discordant with our hypothesis, as well as a Canadian surgical study and an Australian workers’ compensation study.^11,14^ The association of sprain and strain injuries with persistent opioid use has been previously reported.^11^ However, to our knowledge, the association between crush injuries and persistent opioid use is a unique finding.

The strong association between persistent opioid use and chronic pain diagnoses are concerning and may highlight a critical gap between national evidence-based guidelines and actual prescribing practices. However, it should be noted that the cohort could not discern from the claims data when the chronic pain condition was first diagnosed, and was unable to comment on what proportion of these conditions existed prior to the injury. It is possible that some participants sought a chronic pain diagnosis to justify a continued disability claim. In addition, the cohort did not have data on the use of nonopioid pain management therapies. Evaluating the effectiveness of alternative pain management strategies in lowering pain and opioid reliance in the workers’ compensation population is an important area for future research.

### Limitations

This study presents a large workers’ compensation sample with 9 years of data. The use of claims records provides a robust and complete data source. Despite the strengths of this study, the findings must be interpreted within the limitations of the available data. The dose and the duration for the prescribed opioid were infrequently reported and therefore we were unable to include those data in the analysis. It is therefore possible to have a gap in opioid use during the 90-, 180-, and 365-day time intervals. Data were only available on the study participants from the time of their workers’ compensation claim, and the participant’s previous medication and medical history were not available. In addition, it is likely that many study participants had additional health insurance protection outside of their workers’ compensation coverage. Some medical expenses, including opioid prescriptions, may have been reimbursed from other sources and would not have been included in our analysis. Furthermore, the opioid claims data only determine if the opioid prescription was filled. The actual use of the opioid cannot be ascertained from the available data. All of these issues lead to imperfect specificity in our primary outcome. However, we assume the outcome misclassification to be nondifferential, therefore resulting in an underestimation of the OR associated with the included factors. The study only included mental health diagnoses that were included with the workers’ compensation claim and therefore assumes the proportion of the sample with previous diagnosis of conditions such as depression, anxiety, and other mood disorders that have been previously identified to be associated with persistent opioid use in other studies to be underreported in these data.^15,28,29^ The characteristics of the prescribing clinician were also not available, preventing the assessment of divergent prescribing practices by specialty and individual clinicians. The type of injury was determined using *ICD-9* codes, providing limited mechanistic information and the potential for coding error.

Persistent opioid use bears significant risks and costs with insufficient evidence to support their effectiveness as a pain management therapy.^30,31^ This large retrospective cohort study provides important insight into the factors associated with persistent opioid use among workers’ compensation patients. Factors identified in these data should be closely monitored by clinicians, insurance professionals, and employers. To curb the opioid epidemic and prevent the untimely deaths of scores of Americans, policies to support alternative pain management therapies that minimize opioid therapy should be investigated, particularly for high-risk patients identified in this study with nonspecific diagnoses such as strain or sprain injuries.

## Conclusions

Of the workers’ compensation claimants in Maryland with an initial opioid prescription, 28.6% filled a subsequent opioid prescription more than 90 days from injury. Persistent opioid use was determined to be most significantly associated with increased age, strain and sprain injuries, crush injuries, annual income of at least $60 000, claims adjudicated as permanent total disability, and the concomitant diagnoses of chronic joint pain and other chronic pain such as migraines and fibromyalgia. Interventions to lower persistent opioid use in this population should target patients with these identified characteristics.

## Notes

Supplement. **eTable.** *ICD-9* Codes Used to Determine Chronic Pain Diagnosis **eReference**
