# Supplementary material for: Factors Associated With Persistent Opioid Use Among Injured Workers’ Compensation Claimants
Source: JAMA Netw Open. 2018 Oct 26;1(6):e184050. doi: 10.1001/jamanetworkopen.2018.4050 (PMC6324441; doi:10.1001/jamanetworkopen.2018.4050)
Supplement: Supplement. — eTable. ICD-9 Codes Used to Determine Chronic Pain Diagnosis eReference [file jamanetwopen-1-e184050-s001.pdf]

Supplementary Online Content

O'Hara NN, Pollak AN, Welsh CJ, et al. Factors associated with persistent opioid use among injured workers' compensation claimants. *JAMA Netw Open*. 2018;1(6):e184050. doi:10.1001/jamanetworkopen.2018.4050

eTable. *ICD-9* Codes Used to Determine Chronic Pain Diagnosis
eReference

This supplementary material has been provided by the authors to give readers additional information about their work.

© 2018 O'Hara NN et al. *JAMA Network Open*.

eTable. *ICD-9* Codes Used to Determine Chronic Pain Diagnosis

**Pain Disorder Category**	**ICD9 Code**
Back	722.30, 722.32, 722.33, 722.70, 722.72, 722.73, 722.80, 722.82, 722.83, 722.90, 722.92, 722.93, 737.1, 737.3, 738.4, 738.5, 739.2, 739.3, 739.4, 756.10, 756.11, 756.12, 756.19, 805.4, 805.8, 839.2, 839.42, 846, 846.0, 847.1, 847.2, 847.3, 847.9, 721.3x - 721.9x, 722.2x, 724.xx, 756.13
Neck	721.0X, 721.1X, 722.0X, 722.31, 722.71, 722.81, 722.91, 723.XX, 839.0, 839.1, 847.0
Arthritis and joint pain	711.XX, 712.XX, 713.X, 714.XX, 715.XX, 716.XX, 717.XX, 718.XX, 719.XX, 725, 726.XX, 727.XX, 728.XX, 729.3X, 729.7X, 729.8X, 729.9X, 730.XX, 731.X, 732.X, 733.XX, 734, 735.X, 736.XX, 737.2X, 737.4X, 738.1X, 710, 710.1, 710.3, 710.4, 710.5, 710.8, 710.9, 729, 729.2, 729.4, 729.5, 729.6, 737, 737.8, 737.9, 738, 738.2, 738.3, 738.6, 738.7, 738.8, 738.9, 739, 739.1, 739.5, 739.6, 739.7, 739.8, 739.9
**Other Pain Disorders**	
TMJD	524.60, 524.61, 524.62, 524.63, 524.64, 524.69
Tension headache	307.81
Headache/head pain/suboccipital headache	784.0
Occipital neuralgia/headache	723.8
Tear film insufficiency, unspecified (dry eyes)	375.15
Myalgia and myositis, unspecified (Fibromyalgia)	729.1
Noncardiac or musculoskeletal chest pain	786.59
Dyskinesis of the esophagus	530.5
Functional bowel (includes IBS)	564.xx
Interstitial cystitis	595.1
Vulvodynia	625.7x
Endometriosis	617.x
Dyspepsia	536.8
Sicca syndrome	710.2
Tinnitus	388.3
Chronic fatigue syndrome	780.71
Migraine	346.00, 346.01, 346.02, 346.03, 346.10, 346.11, 346.12, 346.13, 346.20, 346.21, 346.22, 346.23, 346.30, 346.31, 346.32, 346.33, 346.40, 346.41, 346.42, 346.43, 346.50, 346.51, 346.52, 346.53, 346.60, 346.61, 346.62, 346.63, 346.70, 346.71, 346.72, 346.73, 346.80, 346.81, 346.82, 346.83, 346.90, 346.91, 346.92, 346.93
Insomnia	780.52

© 2018 O’Hara NN et al. *JAMA Network Open*.

eReference

1. Adapted from: Brummett CM, Waljee JF, Goesling J, et al. New persistent opioid use after minor and major surgical procedures in US adults. *JAMA Surg.* 2017 Jun 21;152(6):e170504.

© 2018 O'Hara NN et al. *JAMA Network Open*.
